# Supplementary material for: Dynamic causal brain circuits during working memory and their functional controllability
Source: Nat Commun. 2021 Jun 29;12:3314. doi: 10.1038/s41467-021-23509-x (PMC8241851; doi:10.1038/s41467-021-23509-x)
Supplement: Supplementary file 1 — Supplementary Information [file 41467_2021_23509_MOESM1_ESM.pdf]

Supplementary Information

I. Supplementary Methods

A. Controllability of functional brain networks

Dynamic control processes related to functional brain network organization were modeled as a linear, discrete, time invariant systems of the form

$$\mathbf{x}(k+1) = \mathbf{A}\mathbf{x}(k) + \mathbf{B}\mathbf{u}(k) \qquad \text{(S.1)}$$

$\mathbf{x} \in R^n$ denotes the state of the various brain regions over time. The matrix $\mathbf{A} \in R^{n \times n}$ is a weighted connectivity matrix whose elements describe interactions between individual brain regions. The input matrix $\mathbf{B}$ identifies the control nodes in the brain and is usually of the form $\mathbf{B} = [\mathbf{e}_1 \dots \mathbf{e}_m]$, e_i denotes the n-dimensional i-th canonical vector. $\mathbf{u}(\mathrm{k})$ is the control signal.

Standard results from control theory have shown that a system is controllable if and only if there exists a unique positive definite solution W to the "Lyapunov equation"

$$\mathbf{AWA}' - \mathbf{W} + \mathbf{BB}' = 0. \qquad (S.2)$$

In particular the unique positive solution takes the form

$$\mathbf{W} = \sum_{\boldsymbol{\tau}=\mathbf{0}}^{\infty} \mathbf{A}^{\boldsymbol{\tau}}\mathbf{B}\,\mathbf{B}' \left(\mathbf{A}'\right)^{\boldsymbol{\tau}} \qquad (S.3)$$

This positive definite matrix **W** is often referred to as the controllability Gramian. For practical purposes, in the case of complex systems, the classical notion of controllability, which is a qualitative measure is not sufficient and quantitative measures of controllability are necessary. In particular we are interested in assigning a value to each node, or a set of nodes, which quantifies the influence of each node or set of nodes over the entire network.

We used the average controllability over random target states, which is given by the trace of controllability Gramian $\mathbf{W}$ as our quantitative metric of controllability. For large networks, controllability in all directions of state space is hard to achieve [1]. Moreover, computing other Gramian-based controllability metrics such as $\mathrm{trace}(\mathbf{W}^{-1})$, $\lambda_{min}(\mathbf{W})$ or $\det(\mathbf{W})$ are computationally infeasible due to numerical issues as the size of the network grows. Hence we employ the trace of controllability Gramian as our metric for controllability. This quantity is termed as the average controllability and is inversely related to the average control energy. Nodes or modules with high average controllability are the ones which are expected to have a larger (control-) influence over the network. In terms of the energy a large **W** would mean a smaller amount of energy required to transfer the system to any point in the state space.

$$(S.4)$$

The average controllability, used as the controllability metric in this paper, is defined as

$$\mathbf{W}_{avg} = T(\mathbf{W}) = T\left(\sum_{\boldsymbol{\tau}=\mathbf{0}}^{\infty} \mathbf{A}^{\boldsymbol{\tau}}\mathbf{B}\,\mathbf{B}' \,\left(\mathbf{A}'\;\right)^{\boldsymbol{\tau}}\right) \qquad (S.5)$$

where $T()$ is a trace of a matrix. As a simple illustration consider a causal directed graph with 5 nodes as shown in **Supplementary Figure 1**. For simplicity we assume that the input signal **u** is applied to node 1. Controllability of the network via this input would mean the ability of the node where input of applied (node 1 in this case) to influence (or control) the other nodes (2-5). As we show below, the energy needed to perturb a specific state is directly related to the weighted sum of the out degree. In general, if there is no directed path between node i and node j, then the node j cannot be controlled or influenced by a control input at node i and hence the network is said to be uncontrollable from node i. The network in this example can be controlled by input to any of the 5 nodes. For a complete graph theoretical explanation, we refer to [2].

B. Node-wise controllability

We used multivariate dynamical systems identification (MDSI) (see **Methods**) to compute A, the asymmetric task-dependent causal interactions between brain regions. We then threshold the resulting A matrices for the 2-back and 0-back task conditions to prune out weak connections. Thresholds were chosen such that the resulting connectivity structure captured the strongest and most relevant connections with the constraint of a connected digraph [3].

From the control-theoretic point of view, we were interested in the influence of each node over the rest of the network. This was operationalized by computing the average controllability of each node in the network. We therefore assume that each node has some (control-) influence over all other nodes, that is, the network is controllable individually from each node. Based on this assumption, we threshold the weights of the connectivity matrix, such that the identified system is controllable from each node.

To test the controllability from each node, we employ the eigenvector test [4]. To summarize, our thresholding is based on the following two assumptions:

1. The thresholding should first result in a connected graph, or a graph having one single connected component.
2. Further, the network should be controllable from each node.

C. Network-wise controllability

In addition to determining average controllability associated with each node, an interesting problem is to find the set of input nodes that maximize the trace of the controllability Gramian. In other words, the challenge is to determine the set of best $k < n$ nodes that maximizes the trace of the Gramian. Mathematically, this can be formulated as a set function optimization problem

$$\max_{K \subseteq V} T\big(\mathbf{W}(K)\big), \quad V = \{1, \dots . k\}, \quad |K| = k \leq n \qquad (S.6)$$

Even though one can solve this problem using a brute force method by evaluating the trace across all the possible subsets and pick up the best one, this method becomes unfeasible for large networks. It has been shown that any linear function of the controllability Gramian is a modular set function [5]. This implies that we can choose the best k nodes by individually computing the Gramian for each node and choose the top k nodes with the highest value for the Gramian. In the present study, we use this result to contrast the controllability of SN, FPN and DMN, but in principle this could be used to evaluate arbitrary sets of control nodes.

D. Average controllability in asymmetric networks

Current work on controllability in human brain networks is based on symmetric connections estimated using DTI. However, the connectivity matrix need not be symmetric even in the case of structural connectivity: it is well known that most anatomical connections in the brain are not symmetric [6] , which poses other problems for the application of DTI-based measures in controllability analysis. Crucially, for our purposes here, we show below that under more general conditions in which the connectivity matrix is not symmetric, and with entries not necessarily non negative, average controllability is related to the (absolute values of) weighted out-degree but not the weighted in-degree or the weighted degree. To clarify this, we generated a random 100-node network and examined average controllability in relation to several node degree measures.

We evaluated the relation between average controllability and weighted out-degree. We evaluated the weighted out-degree as (a) the algebraic sum of the outgoing edge weights for each node: $d_i = \sum_{j=1}^{n} \mathbf{A}_{ji}$. (b) sum of the absolute weight of the causal influences: $|d_i| = \sum_{j=1}^{n} |\mathbf{A}_{ji}|$. We found that the average controllability was related to the absolute weighted out-degree ($p<0.001$) but not the signed out-degree ($p=0.94$) (**Supplementary Figure 2**).

Next, we conducted similar analyses examining average controllability vs in-degree. We evaluated the weighted in-degree as (a) the algebraic sum of the incoming edge weights for each node: $d_i = \sum_{j=1}^{n} \mathbf{A}_{ij}$. (b) sum of the absolute weighted incoming causal influences: $|d_i| = \sum_{j=1}^{n} |\mathbf{A}_{ij}|$. We found that the average controllability was not related to either the absolute weighted in-degree ($p=0.85$) or the signed in-degree ($p=0.77$) (**Supplementary Figure 3**).

We then evaluated the relation between average controllability and the difference in (a) the values of the weighted in and out degrees: $dI_i = \sum_{j=1}^{n} \mathbf{A}_{ji} - \sum_{i=1}^{n} \mathbf{A}_{ij}$ and (b) the absolute values of the weighted out and in degrees: $|dI_i| = |\sum_{j=1}^{n} |\mathbf{A}_{ji}| - \sum_{i=1}^{n} |\mathbf{A}_{ij}||$. We found that average controllability was not correlated to either the absolute weighted net difference ($p=0.48$) or the signed weighted net difference ($p=0.86$) (**Supplementary Figure 4**).

These analyses demonstrate that average controllability depends crucially on network asymmetries, and are consistent with the intuition that nodes with the highest outflow also have the highest ability to modulate ongoing neural activity in other brain regions. Finally, we generalized these analyses using 100 random networks of 11 nodes. As in the example above, average controllability was only correlated with absolute sum of the weighted out-degree (**Supplementary Figure 5**).

II. Supplementary Notes

A. Working memory load effects on outflow and inflow weights

Our main analysis focused on net outflow measured as the difference of outflow and inflow weights. Here we extend this analysis to probe working memory load effects on outflow and inflow weights separately. Paired t-tests revelaed that lMFG, rMFG, lFEF, lIPL and DMPFC have significantly greater outflow weights in the 2-back than 0-back (ps<0.05, FDR corrected), rAI and PCC have significantly smaller outflow weights in the 2-back than 0-back (ps<0.05, FDR corrected), and rFEF, rIPL and VMPFC do not have significantly different outflow weights between conditions (**Supplementary Figure 6A**). The SN and FPN had significantly greater outflow in the 2-back than 0-back conditions whereas DMN had significantly weaker outflow in the 2-back than 0-back condition (ps<0.05, FDR corrected) (**Supplementary Figure 6B**). In both 2-back and 0-back conditions, the SN had significantly greater outflow degree than FPN and DMN (ps<0.05, FDR corrected).

We then examined load effects on causal inflow. Paired t-tests showed that lAI, lMFG, rMFG, lFEF, rFEF, lIPL, rIPL, VMPFC and DMPFC have significantly greater inflow weights in the 2-back than 0-back (ps<0.05, FDR corrected), and rAI and PCC do not have significantly different inflow weights between conditions (**Supplementary Figure 6A**). We found that the FPN had significantly greater inflow weights in the 2-back than 0-back condition (ps<0.05, FDR corrected) but SN and DMN did not show significant difference in inflow weights (**Supplementary Figure 6B**). In both 2-back and 0-back condition, FPN had significantly greater inflow weights than SN and DMN (ps<0.05, FDR corrected).

These results demonstrate distinct outflow and inflow weights associated with memory load modulation.

B. Reproducibility of findings in subsamples

To further evaluate the reproducibility of our findings, we split the data into three subsets (N= 245) and replicated the outflow hubs of the rAI (all *ps*<0.05, FDR corrected), the inflow hub of the rMFG (all *ps*<0.05, FDR corrected), the load effect network controllability (all *ps* < 0.001) and high controllability in SN in each subset (all *ps* < 0.001) (**Supplementary Figures 7-9**).

C. Replication of network controllability findings using normalized causal interaction weights

MDSI simultaneously estimates the causality between regions (A matrices) under each condition within the same modeling framework. Therefore, the average controllability estimated under the different conditions can be directly compared without the need for additional normalization. To ensure that our findings were robust with respect to with respect to mean connection strength in

each condition, we conducted network controllability analyses on normalized causal interaction weights. We replicated all our findings as described below.

We repeated the analysis with normalized As. Network controllability was evaluated for each node and task condition and entered into an ANOVA with factors working memory load and node. We found a significant main effect of node ($F_{10,736} = 26.73$, $p < 2.0e\text{-}16$) and load ($F_{1,736} = 316.5$, $p < 2.0e\text{-}16$) (**Supplementary Figure 10A**). Network controllability was lower in the 2-back, compared to the 0-back condition, and this finding held for all nodes (all *ps* < 0.001).

To further evaluate the differential controllability of the three brain networks, we grouped ROIs' controllability scores by their networks and conducted an ANOVA with factors working memory load and network (SN, FPN and DMN). We found a significant main effect of network ($F_{2,736} = 121.8$, $p < 2.0e\text{-}16$), and load ($F_{1,736} = 309.4$, $p < 2.0e\text{-}16$), and a significant interaction between load and network ($F_{2,736} = 28.65$, $p < 6.24e\text{-}13$). SN nodes (rAI, lAI, and DMPFC) had the highest level of controllability in both the 0-back and 2-back tasks. Network controllability was higher in the 0-back, compared to the 2-back condition, and this finding held for all three networks (all *ps* < 0.001, Bonferroni corrected) (**Supplementary Figure 10B**). Further analysis revealed that the load x network interaction arose from higher controllability of the SN compared to the FPN ($t = 4.64$, $p = 4.05e\text{-}06$) and the DMN ($t = 6.46$, $p = 1.82e\text{-}10$), and higher controllability of the FPN compared to DMN ($t = 3.55$, $p = 0.0003$).

We then examined the stability of these findings, focusing first on working-memory load dependent differences in network controllability. Higher network controllability on the 0-back, compared to the 2-back, condition achieved a high level of stability (>80%), with sample sizes of N=30 or more (**Supplementary Figure 10C**). Results showing network differences, with SN having the highest network controllability, also showed a high level of stability (>80%) with samples of N=100 or more for the 2-back task condition and N=50 or more for the 0-back condition.

In summary, we have shown that normalization of directed causal weights in the 0-back and 2-back conditions does not change the main findings.

D. Robustness of findings with respect to meta-analysis defined ROIs

To demonstrate the reproducibility of our findings with respect to node selection, we conducted additional analysis using another set of ROIs determined using meta-analysis of working memory studies. Specifically, we searched "working memory" term in NeuroSynth (https://www.neurosynth.org/), which identified 1,091 studies and 39,905 activations. We selected activation peaks in the lAI, rAI, DMPFC, lMFG, lFEF, lIPL, rMFG, rFEF and rIPL. Because the meta-analysis did not report deactivations, we kept the original PCC and VMPFC ROIs. Each ROI was created using 6-mm radius spheres whose centers are activation peaks. We repeated our analyses with this set of ROIs, and replicated all our main findings as summarized below:

MDSI identified several links that showed significant dynamic causal interactions in the 2-back and 0-back task conditions ($p < 0.01$, FDR-corrected) (**Supplementary Figure 11A**). Next,

leveraging the large sample size of the HCP dataset, we examined the stability of dynamic causal interaction patterns using bootstrapping with subsamples ranging from 20 to 600. We found that dynamic causal interaction patterns achieved a high level of stability ($r > 0.8$) with subsample sizes of N=30 or more (**Supplementary Figure 11B**). These results demonstrate that MDSI reliably estimates dynamic causal interaction patterns associated with both the 2-back and 0-back conditions.

We then computed the outflow degree of each node in each task and participant. The outflow degree is the weighted node degree: averaged outflow weights (all the output connections from a node to all other nodes) *minus* averaged inflow weights (all the input connections to a node from all other nodes). The rAI and lAI showed significant positive outflow in both the 2-back and 0-back conditions, with the rAI showing the highest outflow degree ($p < 0.05$, FDR corrected, **Supplementary Figure 12A**). Stability analysis revealed that this rAI finding was highly stable (> 80%) with sample sizes of N=200 or more (**Supplementary Figure 12B**). That is, the rAI showed the consistently highest outflow, across multiple random subsamples of the data.

Similar to our original analysis, the rMFG showed significant inflow ($p < 0.05$, FDR corrected, **Supplementary Figure 12A**). Stability analysis also revealed that this rMFG finding was highly reliable (> 80%) with subsample sizes of N=200 or more (**Supplementary Figure 12B**). That is, the rMFG showed the consistently highest inflow, across multiple random subsamples of the data.

We next examined whether multivariate patterns of dynamic causal interactions differed between the two task conditions. A support vector machine (SVM) algorithm with 10-fold cross-validation revealed a classification accuracy of 68% ($p < 0.01$, permutation test, **Supplementary Figure 13**). To replicate these findings, we then used Lasso and Elastic-Net Regularized Generalized Linear Models (GLMNET) with a 10-fold cross validation. GLMNET analysis revealed a classification accuracy of 68% ($p < 0.01$, permutation test, **Supplementary Figure 13**).

Next we sought to determine specific links which differ in the strength of dynamic causal interactions between the 2-back and 0-back conditions (all *ps* < 0.01, FDR corrected; **Supplementary Figure 14A**). Increased dynamic causal interactions in the 2-back condition were observed primarily between SN and FPN nodes. In contrast, dynamic causal influences from the PCC node in the DMN on the SN and FPN decreased in the 2-back, compared to the 0-back, condition.

We then examined the stability of working-memory load dependent dynamic causal interaction patterns. We found that dynamic causal interaction patterns achieved a high level of stability ($r > 0.8$) with sample sizes of N=200 or more (**Supplementary Figure 14C**). These results demonstrate that MDSI reliably estimates dynamic causal interaction patterns associated with working memory-load.

We then contrasted the net causal influences of each node between the high and low-load working memory conditions. The lIPL showed significantly greater outflow in the 2-back, compared to the 0-back, task condition ($p < 0.05$, FDR corrected; **Supplementary Figure 14B**)

and the DMPFC had marginally significantly greater outflow in the 2-back than 0-back condition (p=0.08), whereas the PCC showed significantly higher net inflow in the 2-back, compared to the 0-back, condition ($p < 0.05$, FDR corrected; **Supplementary Figure 14B**).

Network controllability was evaluated for each node and task condition and entered into an ANOVA with factors working memory load and node. We found a significant main effect of node ($F_{10,736} = 45.16$, $p < 2.0e\text{-}16$) and load ($F_{1,736} = 24.49$, $p < 9.25e\text{-}07$) (**Supplementary Figure 15A**). Network controllability was lower in the 2-back, compared to the 0-back condition, and this finding held for all nodes (all *ps* < 0.001).

To further evaluate the differential controllability of the three brain networks, we grouped ROIs' controllability scores by their networks and conducted an ANOVA with factors working memory load and network (SN, FPN and DMN). We found a significant main effect of network ($F_{2,736} = 213.7$, $p < 2.0e\text{-}16$), and load ($F_{1,736} = 24.27$, $p < 1.03e\text{-}6$), and a significant interaction between load and network ($F_{2,736} = 6.95$, $p < 0.001$). SN nodes (rAI, lAI, and DMPFC) had the highest level of controllability in both the 0-back and 2-back tasks. Network controllability was higher in the 0-back, compared to the 2-back condition, and this finding held for all three networks (all *ps* < 0.001, Bonferroni corrected) (**Supplementary Figure 15B**). Further analysis revealed that the load x network interaction arose from load differences characterized by higher controllability of the SN compared to the FPN ($t = 1.89$, $p = 0.05$) and the DMN ($t = 3.23$, $p = 0.001$), and higher controllability of the FPN compared to DMN ($t = 2.17$, $p = 0.03$).

We then examined the stability of these findings, focusing first on working-memory load dependent differences in network controllability. Higher network controllability on the 0-back, compared to the 2-back, condition achieved a high level of stability (>80%), with sample sizes of N=200 or more (**Supplementary Figure 15C**). Results showing network differences, with SN having the highest network controllability, also showed a high level of stability (>80%) with samples of N=100 or more for the 2-back task condition and N=150 or more for the 0-back condition.

Finally, we investigated whether dynamic causal interactions between the SN, FPN and DMN are related to working memory performance. CCA model fits were significant in the 2-back condition (*Pillai's trace* = 0.37, $p = 0.004$) but not in the 0-back condition (*Pillai's trace* = 0.28, $p = 0.7$). CCA identified a significant relation between dynamic causal weights and behavioral scores in the 2-back condition ($r = 0.47$, $p < 0.001$, **Supplementary Figure 16A**). **Supplementary Figure 16B** illustrates the canonical correlation coefficients and highlights positive influences between SN and FPN nodes and negative influences of SN and FPN nodes on PCC and VMPFC nodes of the DMN.

In summary, these results demonstrate that the entire set of originally reported findings were replicated with the new set of ROIs, the only exception was that between-condition differences in net causal influence from the revised DMPFC node was relatively weaker.

III. Supplementary Figures

Supplementary Figure 1. Directed network with 5 nodes illustrating that input u to node 1 can control all other network nodes.

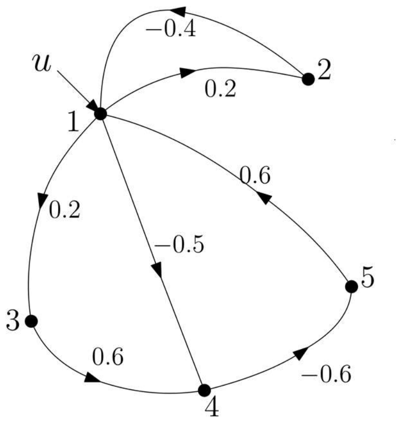

Supplementary Figure 2. Average controllability in relation to Out Degree in simulated asymmetric networks. (Left) Average controllability is not correlated with the weighted out-degree (p=0.94). (Right) However, average controllability is strongly correlated with the absolute weighted Out-degree (p<0.001).

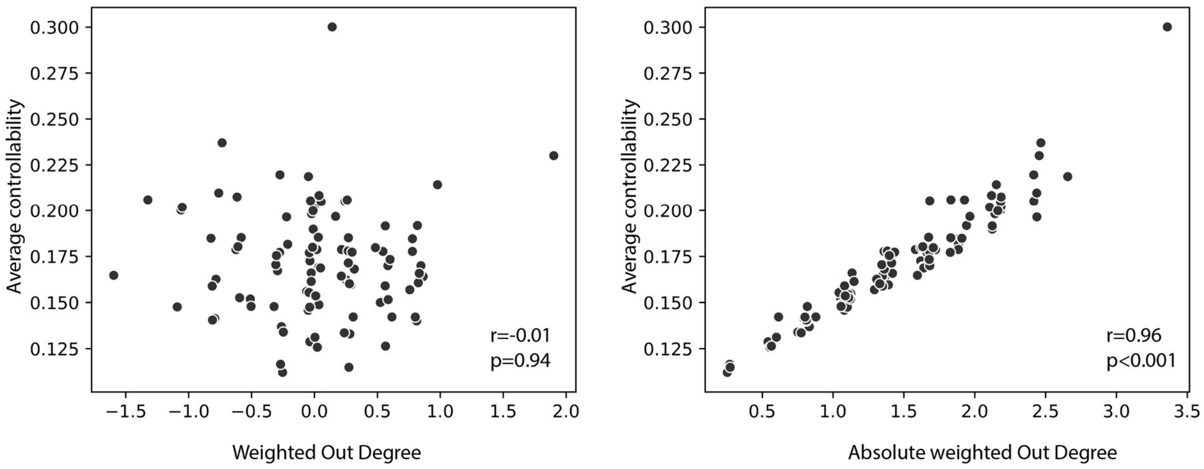

Supplementary Figure 3. Average controllability in relation to In Degree in simulated asymmetric networks. (Left) Average controllability is not correlated with the weighted In-degree (p=0.77). (Right) Average controllability is also not correlated the absolute weighted In-degree (p=0.85).

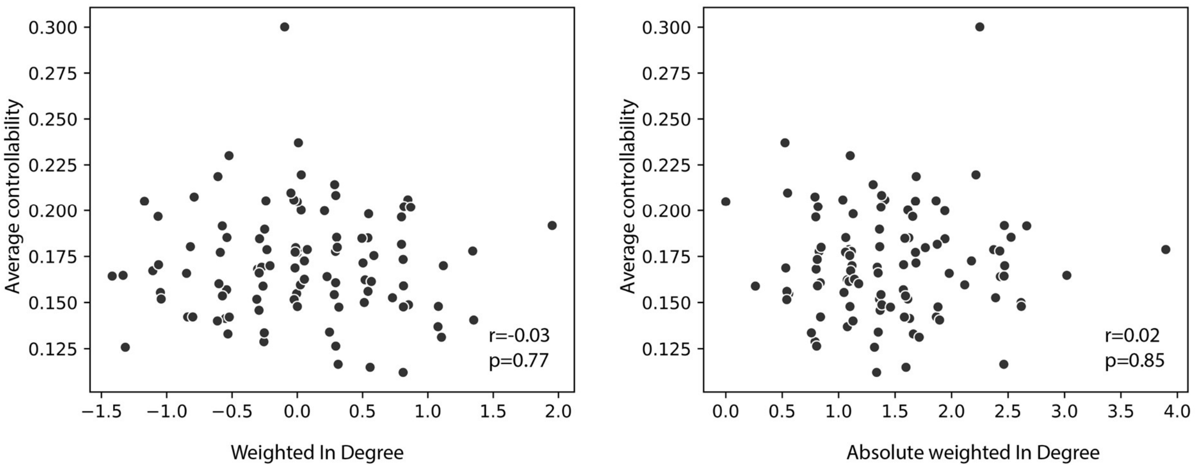

Supplementary Figure 4. Average controllability in relation to Out-In Degree in simulated asymmetric networks. (Left) Average controllability is not correlated with weighted net Out-In degree (p=0.86). (Right) Average controllability is also not correlated with absolute values of weighted net Out-In degree (p=0.48).

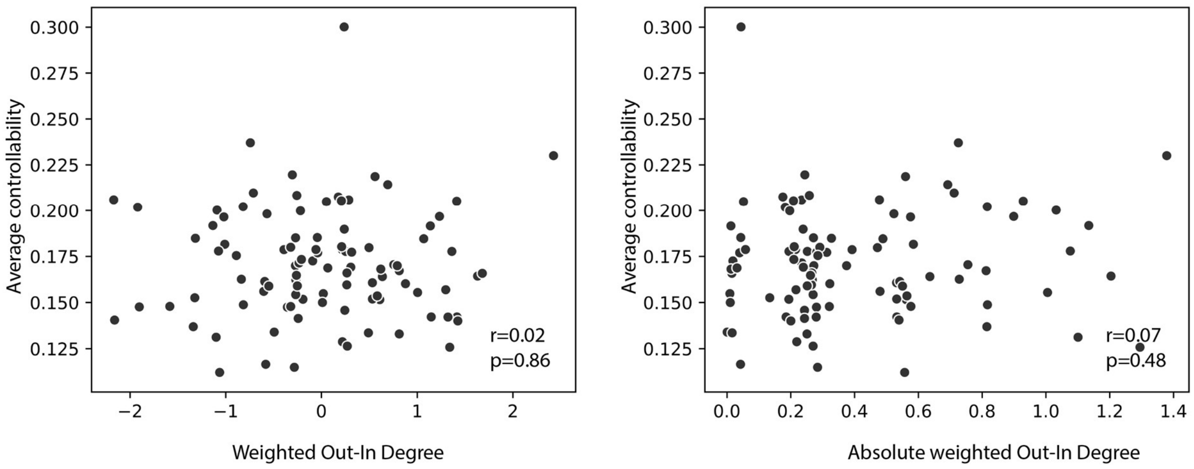

Supplementary Figure 5. Average controllability in relation to network degree in 100 random simulated asymmetric networks with 11 nodes**.** 1: weighted-out degree, 2: absolute weighted-out degree, 3: weighted-in degree, 4: absolute weighted-in degree, 5: difference between absolute weighted out – in degree. Data are presented as mean ± SEM.

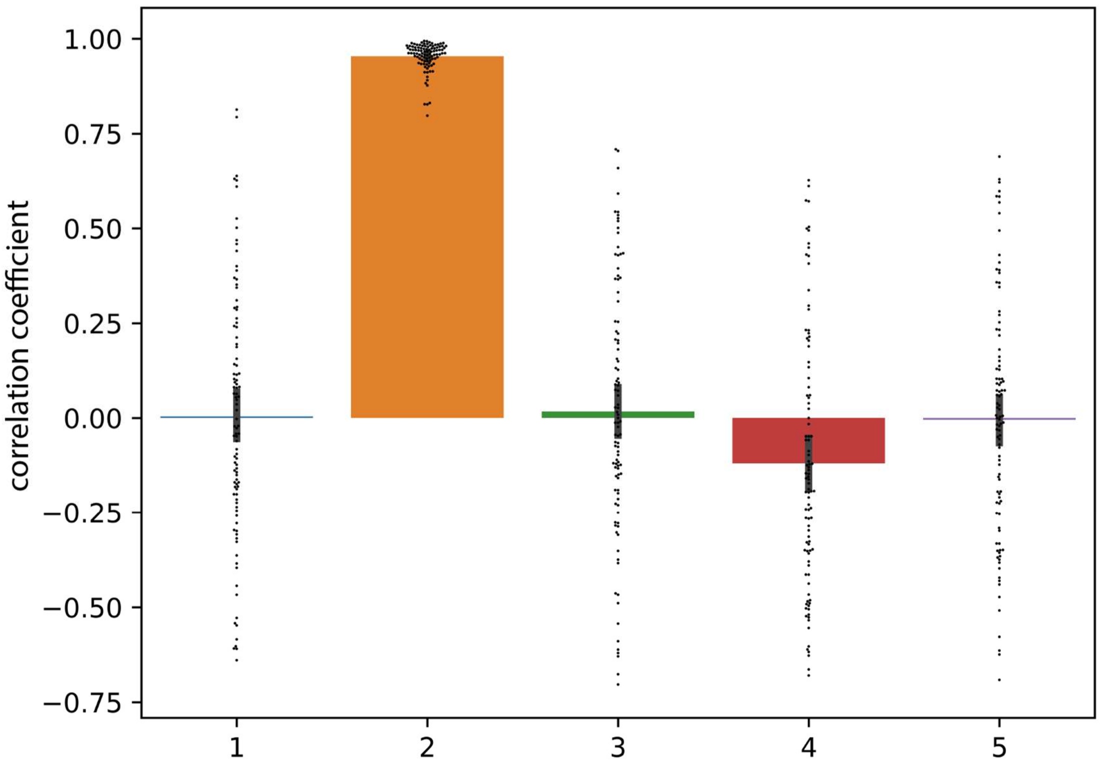

Supplementary Figure 6. Network analysis examined load effects on outflow and inflow weights separately. (A) The lMFG, rMFG, lFEF, lIPL and DMPFC have significantly greater outflow weights in the 2-back than 0-back whereas rAI and PCC have significantly smaller outflow weights in the 2-back than 0-back (ps<0.05, FDR corrected, two-sided t-test). The lAI, lMFG, rMFG, lFEF, rFEF, lIPL, rIPL, VMPFC and DMPFC have significantly greater inflow weights in the 2-back than 0-back (ps<0.05, FDR corrected, two-sided t-test). n = 737 participants. Data are presented as mean ± SEM. (**B**) The SN and FPN had significantly greater outflow weights in the 2-back than 0-back conditions whereas DMN had significantly smaller outflow weights in the 2-back than 0-back condition (ps<0.05, FDR corrected, two-sided t-test). The FPN had significantly greater inflow weights in the 2-back than 0-back condition (ps<0.05, FDR corrected). n = 737 participants. Data are presented as mean ± SEM. lAI: left anterior insula; rAI: right anterior insula; DMPFC: dorsomedial prefrontal cortex; lMFG: left middle frontal gyrus; rMFG, right middle frontal gyrus; lFEF, left frontal eye field; rFEF, right frontal eye field; lIPL, left intraparietal lobule; rIPL, right intraparietal lobule; PCC, posterior cingulate cortex and VMPFC, ventromedial prefrontal cortex; SN, salience network; FPN: frontoparietal network; DMN: default mode network. Source data are provided as a Source Data file.

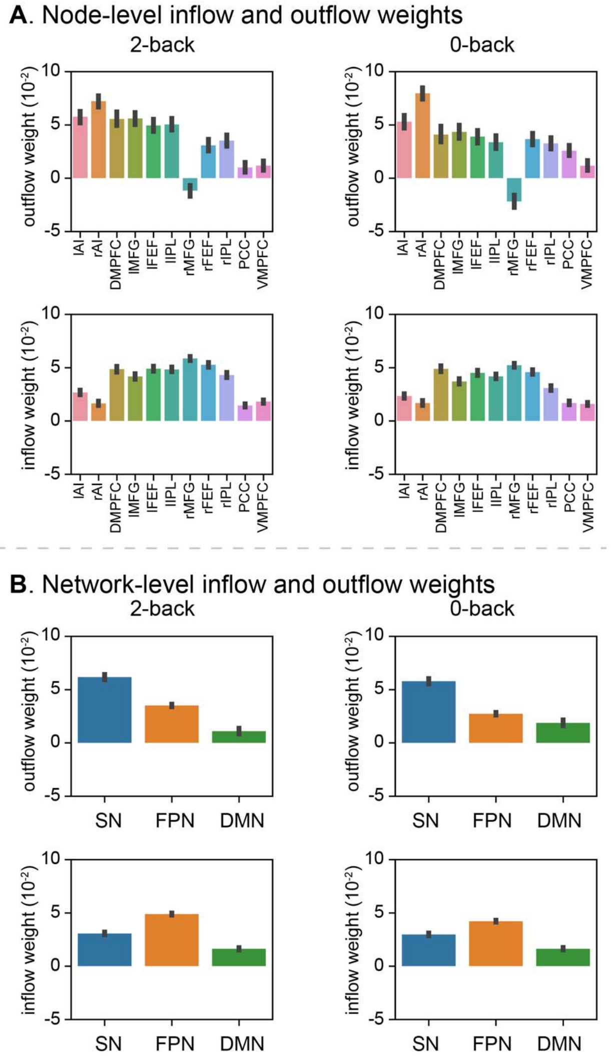

Supplementary Figure 7. Replication of the outflow hub of the rAI and the inflow hub of the rMFG among the ROIs of the SN, FPN and DMN in both 2-back and 0-back task conditions from three subsets (ps<0.05, FDR corrected, two-sided t-test). n = 245 participants. Data are presented as mean ± SEM. lAI: left anterior insula; rAI: right anterior insula; DMPFC: dorsomedial prefrontal cortex; lMFG: left middle frontal gyrus; rMFG, right middle frontal gyrus; lFEF, left frontal eye field; rFEF, right frontal eye field; lIPL, left intraparietal lobule; rIPL, right intraparietal lobule; PCC, posterior cingulate cortex and VMPFC, ventromedial prefrontal cortex. Source data are provided as a Source Data file.

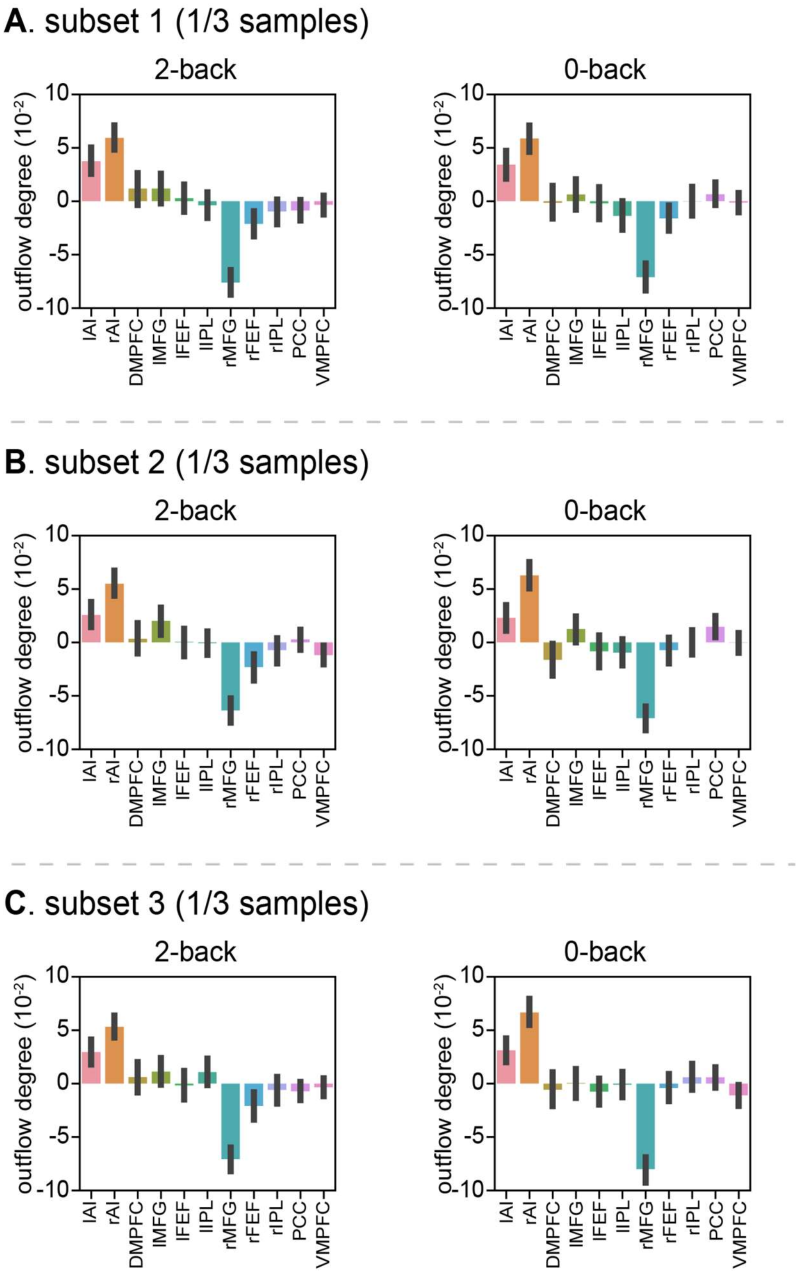

Supplementary Figure 8. Replication of load effect of the controllability in three subsets (ps<0.05, FDR corrected, two-sided t-test). n = 245 participants. Data are presented as mean ± SEM. lAI: left anterior insula; rAI: right anterior insula; DMPFC: dorsomedial prefrontal cortex; lMFG: left middle frontal gyrus; rMFG, right middle frontal gyrus; lFEF, left frontal eye field; rFEF, right frontal eye field; lIPL, left intraparietal lobule; rIPL, right intraparietal lobule; PCC, posterior cingulate cortex and VMPFC, ventromedial prefrontal cortex. Source data are provided as a Source Data file.

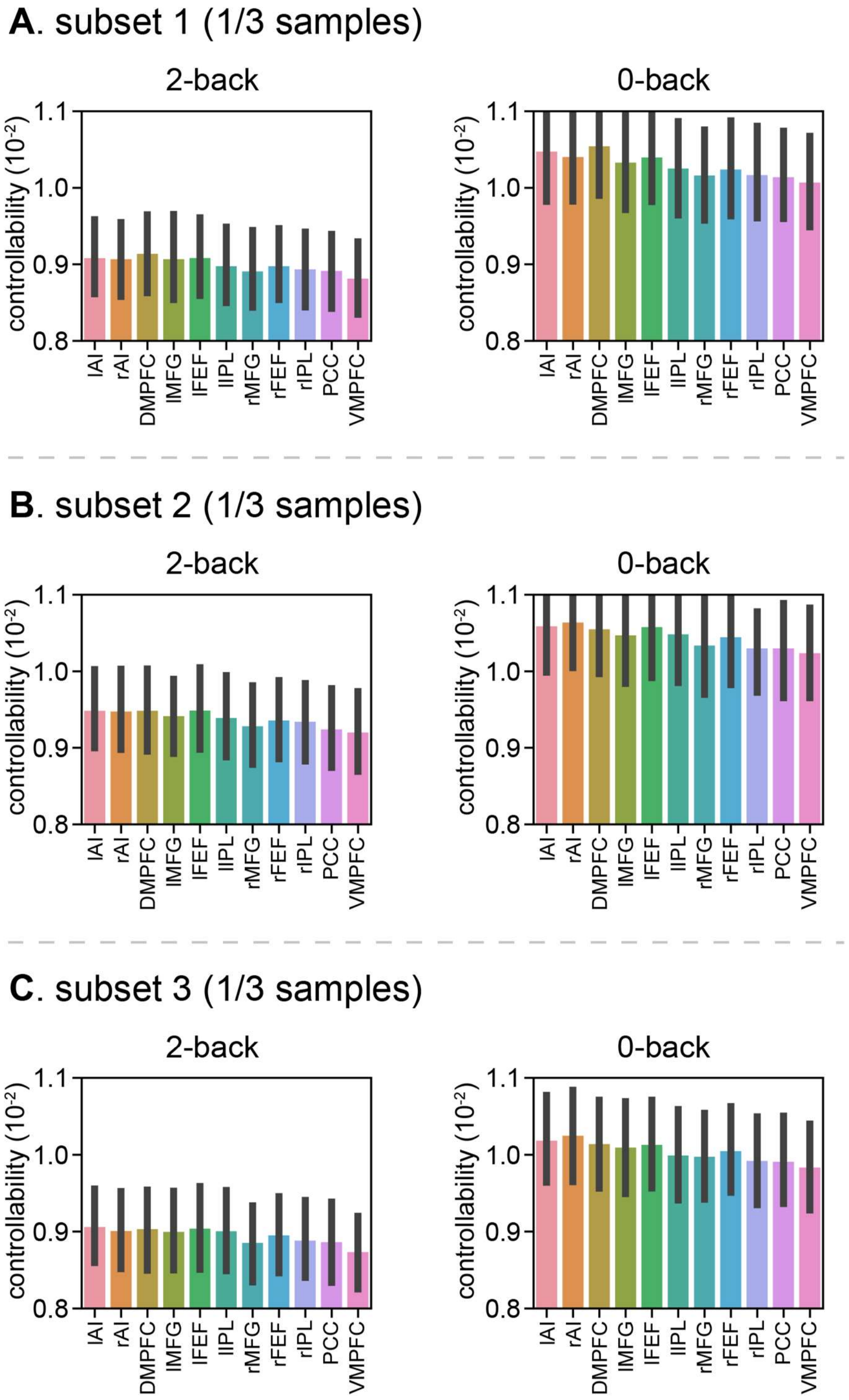

Supplementary Figure 9. Replication of the highest controllability of SN in three subsets (ps<0.05, FDR corrected, two-sided t-test). n = 245 participants. Data are presented as mean ± SEM. SN, salience network; FPN: frontoparietal network; DMN: default mode network. Source data are provided as a Source Data file.

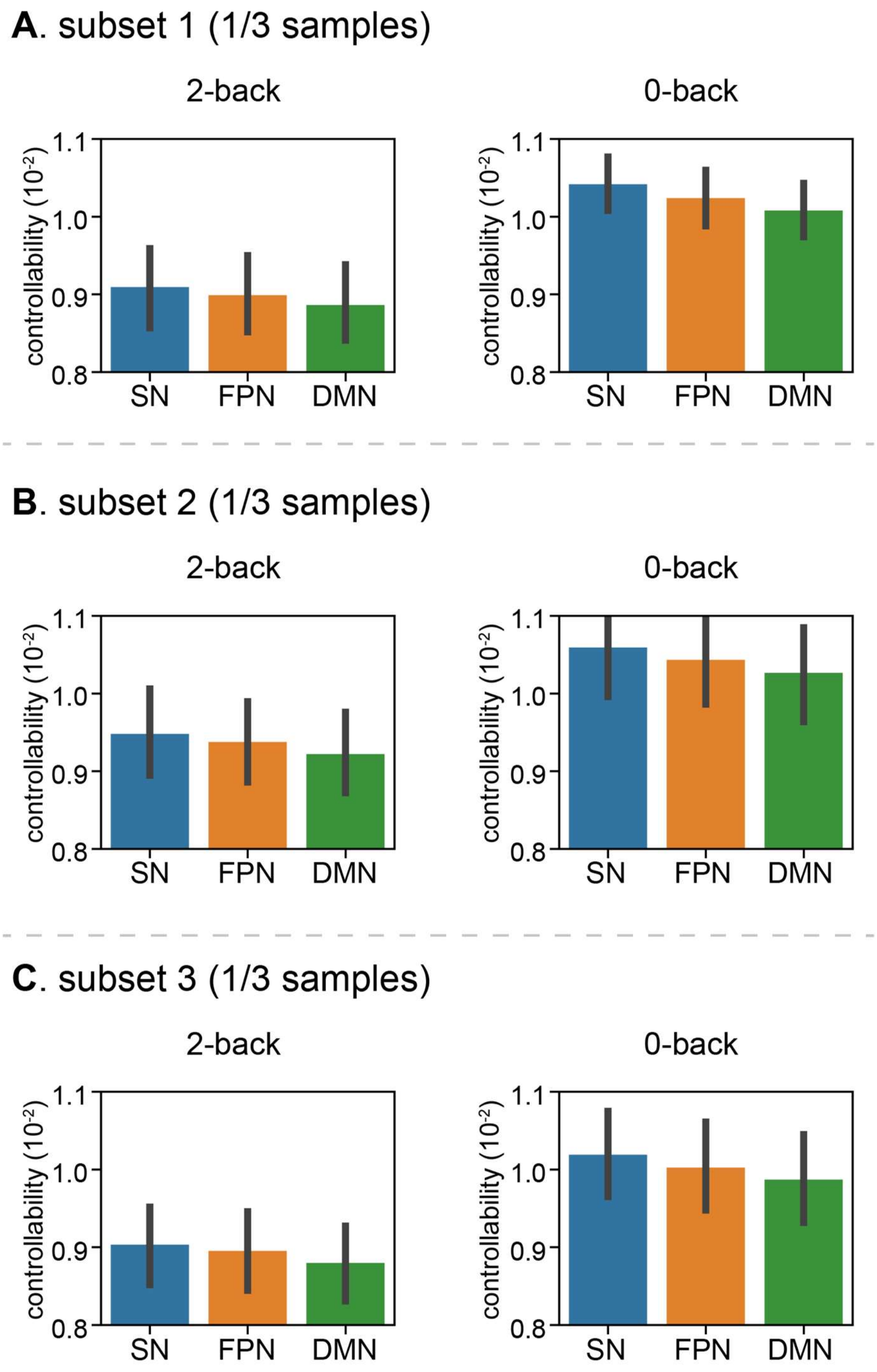

Supplementary Figure 10. Controllability analysis based on normalized MDSI values. (**A**) Functional network controllability, assessed in each brain node, was significantly lower in the 2-back, compared to the 0-back, working memory task condition ($p < 0.001$, two-sided t-test). AI and DMPFC nodes in the SN have significantly higher controllability than FPN and DMN nodes, except for lFEF and lMFG nodes of the FPN in the 2-back task condition and lFEF in the 0-back task condition ($p < 0.001$, two-sided t-test). n = 737 participants. Data are presented as mean ± SEM. (**B**) Functional network controllability, assessed across SN, FPN and DMN nodes, was significantly lower in the 2-back, compared to the 0-back, working memory task condition ($p < 0.001$, two-sided t-test). The SN shows significantly higher controllability than the FPN and DMN ($p < 0.001$, two-sided t-test). n = 737 participants. Data are presented as mean ± SEM. (**C**) Stability analyses revealed stable load effect (0-back > 2-back) and network difference (SN > FPN and SN > DMN). X-axis shows sample size, ranging from 20 to 600. Y-axis shows stability, computed as the probability that the load effect of controllability is significantly different between 2-back and 0-back working memory conditions, and the probability that the SN shows greater controllability than the FPN and DMN in both 2-back and 0-back working memory conditions, in random subsamples drawn from N=737 participants. lAI: left anterior insula; rAI: right anterior insula; DMPFC: dorsomedial prefrontal cortex; lMFG: left middle frontal gyrus; rMFG, right middle frontal gyrus; lFEF, left frontal eye field; rFEF, right frontal eye field; lIPL, left intraparietal lobule; rIPL, right intraparietal lobule; PCC, posterior cingulate cortex and VMPFC, ventromedial prefrontal cortex; SN, salience network; FPN: frontoparietal network; DMN: default mode network. Source data are provided as a Source Data file.

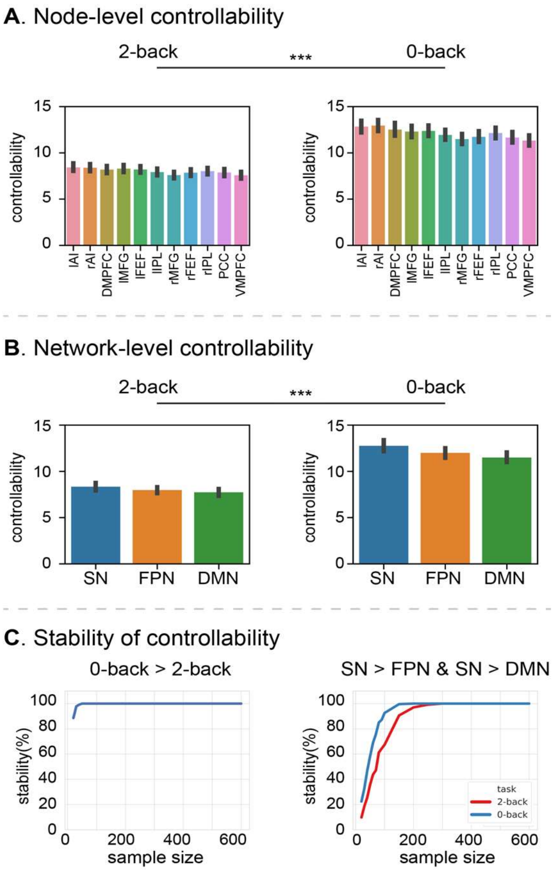

Supplementary Figure 11. Working memory load-specific dynamic causal influences (ROIs determined from NeuroSynth meta-analysis). (**A**) Significant directed causal influences between SN, FPN and DMN ROIs in the 2-back and 0-back working memory task conditions ($p < 0.01$, FDR corrected, two-sided t-test). Red cells indicate significant positive influences and blue indicates significant negative influences. n = 737 participants. (**B**) Stability analyses revealed highly stable multivariate patterns of causal influences among SN, FPN and DMN nodes in 2-back and 0-back task conditions ($r > 0.8$ for Sample size > 25). X-axis shows sample sizes ranging from 20 to 600. Y-axis shows stability, computed as the correlation of multivariate causal influence patterns between the original sample and random subsamples drawn from N=737 participants. lAI: left anterior insula; rAI: right anterior insula; DMPFC: dorsomedial prefrontal cortex; lMFG: left middle frontal gyrus; rMFG, right middle frontal gyrus; lFEF, left frontal eye field; rFEF, right frontal eye field; lIPL, left intraparietal lobule; rIPL, right intraparietal lobule; PCC, posterior cingulate cortex and VMPFC, ventromedial prefrontal cortex. Source data are provided as a Source Data file.

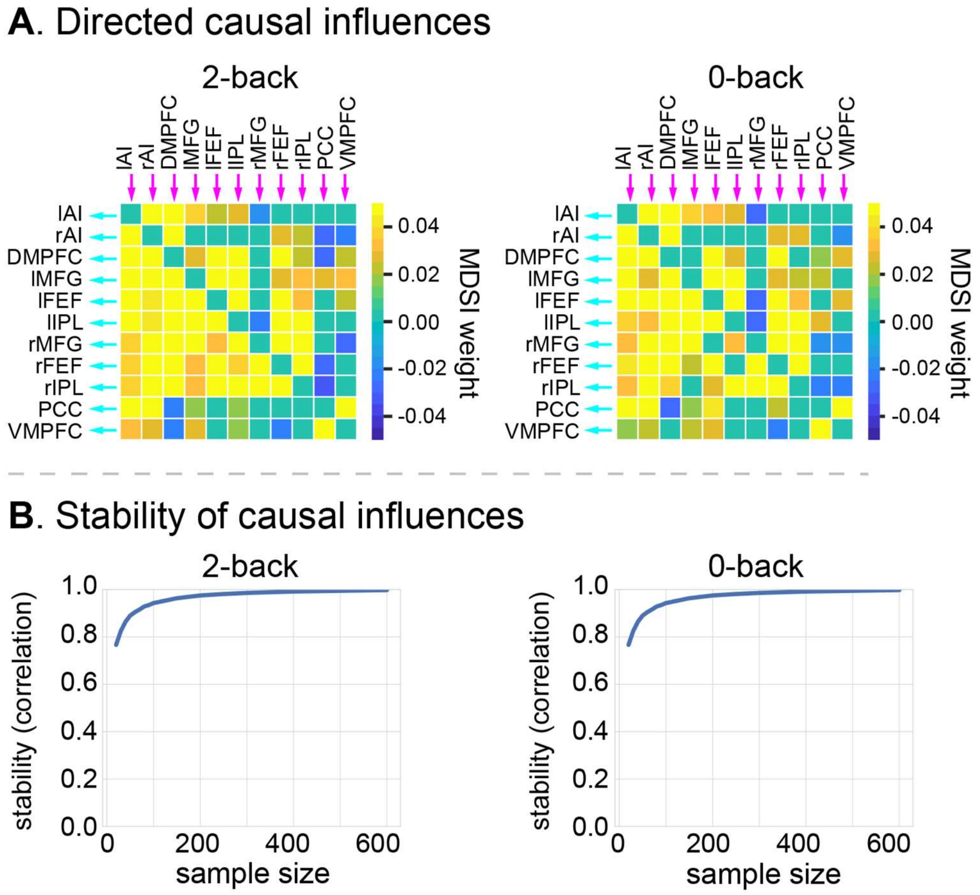

.

Supplementary Figure 12. Working memory load-specific dynamic causal outflow (ROIs determined from NeuroSynth meta-analysis). (**A**) AI showed the highest directed causal outflow between SN, FPN and DMN nodes in both the 2-back and 0-back working memory task conditions ($p < 0.05$, FDR corrected, two-sided t-test). In contrast, the rMFG showed the highest directed causal inflow among all nodes in both task conditions ($p < 0.05$, FDR corrected, two-sided t-test). n = 737 participants. Data are presented as mean ± SEM. **(B)** Stability analyses revealed highly stable directed causal outflow from the rAI and directed causal inflow into the rMFG in both the 2-back and 0-back working memory task conditions. X-axis shows sample size, ranging from 20 to 600. Y-axis shows stability, computed as the probability that the rAI shows the highest positive directed causal outflow among SN, FPN and DMN nodes, and the probability that the rMFG shows the highest causal inflow in random subsamples drawn from N=737 participants. lAI: left anterior insula; rAI: right anterior insula; DMPFC: dorsomedial prefrontal cortex; lMFG: left middle frontal gyrus; rMFG, right middle frontal gyrus; lFEF, left frontal eye field; rFEF, right frontal eye field; lIPL, left intraparietal lobule; rIPL, right intraparietal lobule; PCC, posterior cingulate cortex and VMPFC, ventromedial prefrontal cortex. Source data are provided as a Source Data file.

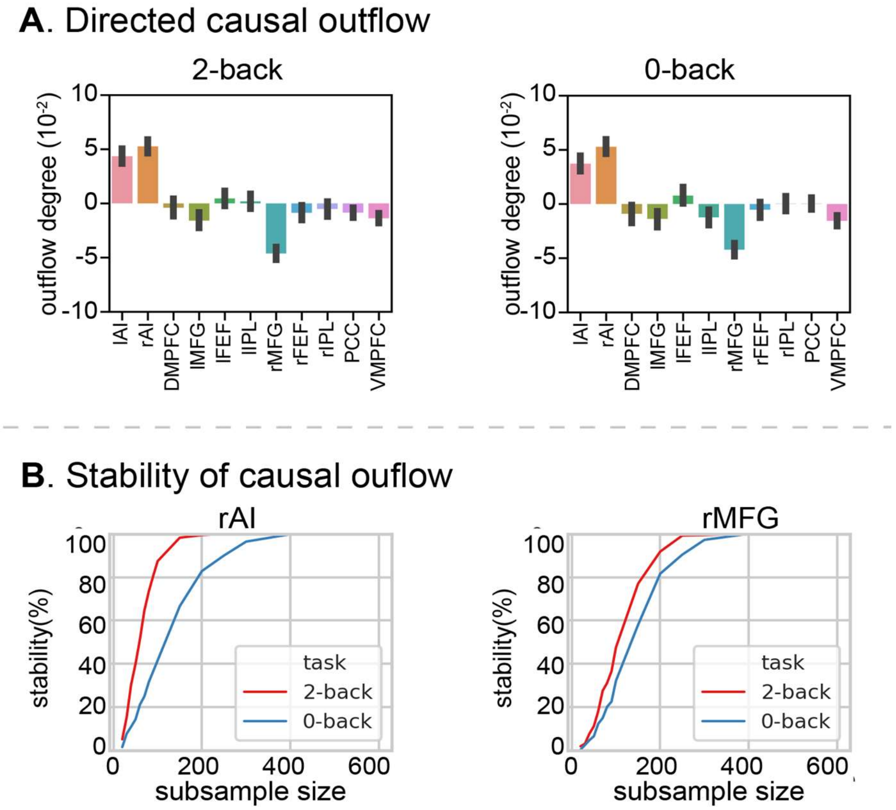

Supplementary Figure 13. Dynamic causal influences distinguish working memory conditions (ROIs determined from NeuroSynth meta-analysis). Both linear SVM and GLMNET analyses with 10-fold cross validation revealed that dynamic causal influences between SN, FPN and DMN nodes distinguished the 2-back and 0-back working memory task conditions (*ps* < 0.01, permutation tests). n = 737 participants. Data are presented as mean ± SEM.

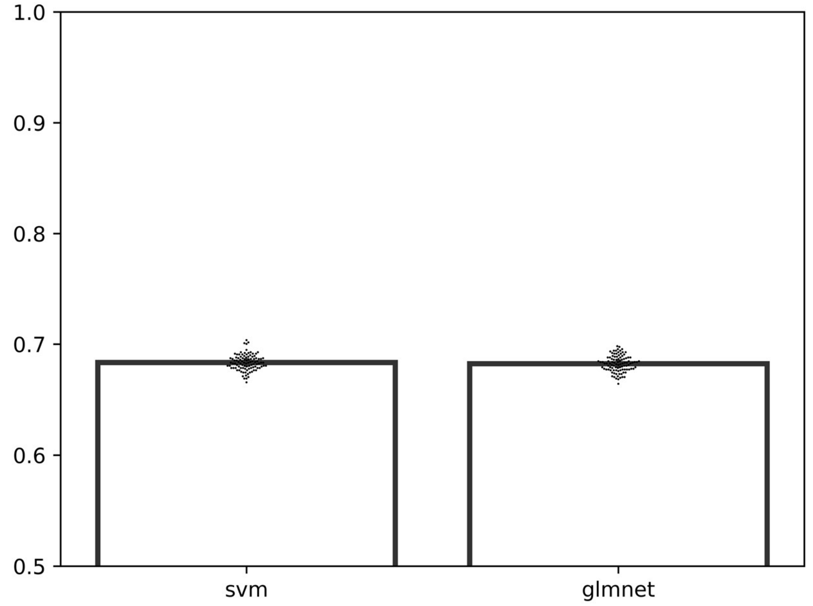

Supplementary Figure 14. Working memory load-dependent dynamic causal influence and net outflow (ROIs determined from NeuroSynth meta-analysis). (**A**) MDSI analysis revealed links with significantly greater directed causal influences between SN, FPN and DMN nodes in the 2-back, compared to the 0-back, working memory task condition ($p < 0.05$, FDR corrected, two-sided t-test). n = 737 participants. (**B**) The lAI, DMPFC and lIPL showed significantly higher directed causal outflow in the 2-back, compared to the 0-back, task condition. In contrast, the PCC showed significantly higher directed causal inflow in the 2-back, compared to the 0-back, task condition ($p < 0.05$, FDR corrected, two-sided t-test). n = 737 participants. Data are presented as mean ± SEM. (**C**) Stability analyses revealed highly stable multivariate patterns of causal influences between SN, FPN and DMN nodes in 2-back versus 0-back ($r > 0.8$ with samples > 200). X-axis is the subsample sizes, ranging from 20 to 600. Y-axis is the stability measures, which is the correlation of multivariate causal interaction patterns between subsamples and original dataset. lAI: left anterior insula; rAI: right anterior insula; DMPFC: dorsomedial prefrontal cortex; lMFG: left middle frontal gyrus; rMFG, right middle frontal gyrus; lFEF, left frontal eye field; rFEF, right frontal eye field; lIPL, left intraparietal lobule; rIPL, right intraparietal lobule; PCC, posterior cingulate cortex and VMPFC, ventromedial prefrontal cortex. Source data are provided as a Source Data file.

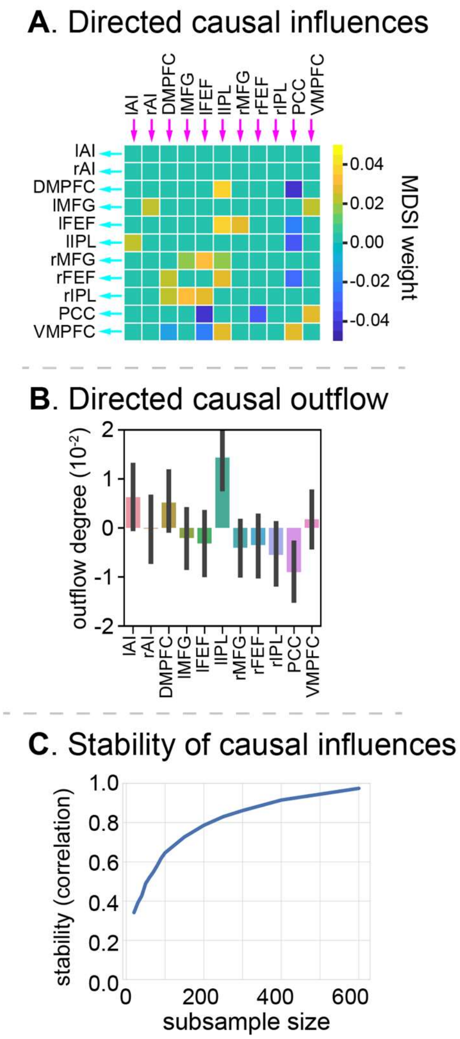

Supplementary Figure 15. Working memory load-dependent functional controllability in SN, FPN and DMN (ROIs determined from NeuroSynth meta-analysis). (**A**) Functional network controllability, assessed in each brain node, was significantly lower in the 2-back, compared to the 0-back, working memory task condition ($p < 0.001$, two-sided t-test). AI and DMPFC nodes in the SN have significantly higher controllability than FPN and DMN nodes, except for lFEF and lMFG nodes of the FPN in the 2-back task condition and lFEF in the 0-back task condition ($p < 0.001$, two-sided t-test). n = 737 participants. Data are presented as mean ± SEM. (**B**) Functional network controllability, assessed across SN, FPN and DMN nodes, was significantly lower in the 2-back, compared to the 0-back, working memory task condition ($p < 0.001$, two-sided t-test). The SN shows significantly higher controllability than the FPN and DMN ($p < 0.001$, two-sided t-test). n = 737 participants. Data are presented as mean ± SEM. (**C**) Stability analyses revealed stable load effect (0-back > 2-back) and network difference (SN > FPN and SN > DMN). X-axis shows sample size, ranging from 20 to 600. Y-axis shows stability, computed as the probability that the load effect of controllability is significantly different between 2-back and 0-back working memory conditions, and the probability that the SN shows greater controllability than the FPN and DMN in both 2-back and 0-back working memory conditions, in random subsamples drawn from N=737 participants. lAI: left anterior insula; rAI: right anterior insula; DMPFC: dorsomedial prefrontal cortex; lMFG: left middle frontal gyrus; rMFG, right middle frontal gyrus; lFEF, left frontal eye field; rFEF, right frontal eye field; lIPL, left intraparietal lobule; rIPL, right intraparietal lobule; PCC, posterior cingulate cortex and VMPFC, ventromedial prefrontal cortex. SN: salience network; FPN: frontoparietal network; DMN: default mode network. Source data are provided as a Source Data file.

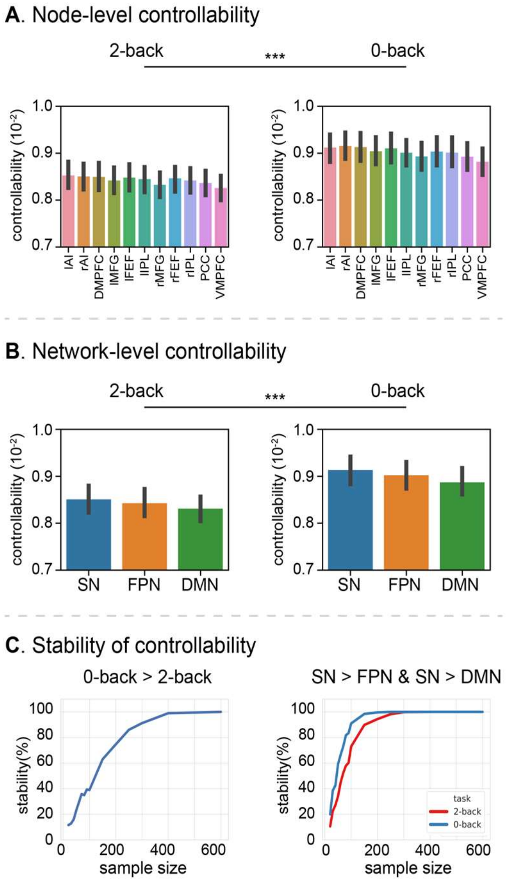

Supplementary Figure 16. Dynamic causal influence relation to behavioral performance (ROIs determined from NeuroSynth meta-analysis). (**A**) Canonical correlation analysis revealed a significant relationship between directed SN, FPN and DMN causal influences and behavioral performance in the 2-back working memory task condition ($r = 0.47$, $p < 0.001$, *Pearson*'s correlation). n = 737 participants. (**B**) Correlation coefficients contributing to brain-behavior relations highlights positive influences between SN and FPN nodes and negative influences of SN and FPN nodes on PCC and VMPFC nodes of the DMN. lAI: left anterior insula; rAI: right anterior insula; DMPFC: dorsomedial prefrontal cortex; lMFG: left middle frontal gyrus; rMFG, right middle frontal gyrus; lFEF, left frontal eye field; rFEF, right frontal eye field; lIPL, left intraparietal lobule; rIPL, right intraparietal lobule; PCC, posterior cingulate cortex and VMPFC, ventromedial prefrontal cortex. Source data are provided as a Source Data file.

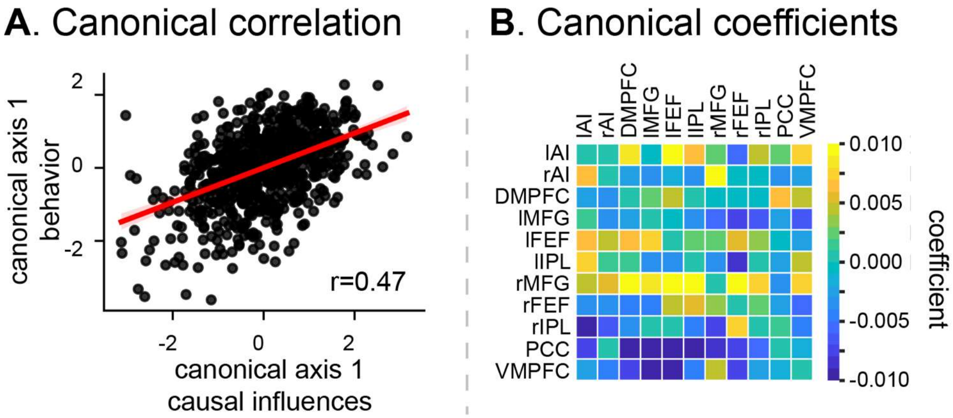

Supplementary Figure 17. Salience Network (SN), Frontal-Parietal Network (FPN) and Default Mode network (DMN) ROIs: 1, left anterior insula (lAI); 2, right anterior insula (rAI); 3, dorsomedial prefrontal cortex (DMPFC); 4, left middle frontal gyrus (lMFG); 5, right middle frontal gyrus (rMFG); 6, left frontal eye field (lFEF); 7, right frontal eye field (rFEF); 8, left intraparietal lobule (lIPL); 9, right intraparietal lobule (rIPL); 10, posterior cingulate cortex (PCC) and 11, ventromedial prefrontal cortex (VMPFC). ROIs' sizes are proportionally to their node degrees in (**A**) 2-back and (**B**) 0-back task conditions.

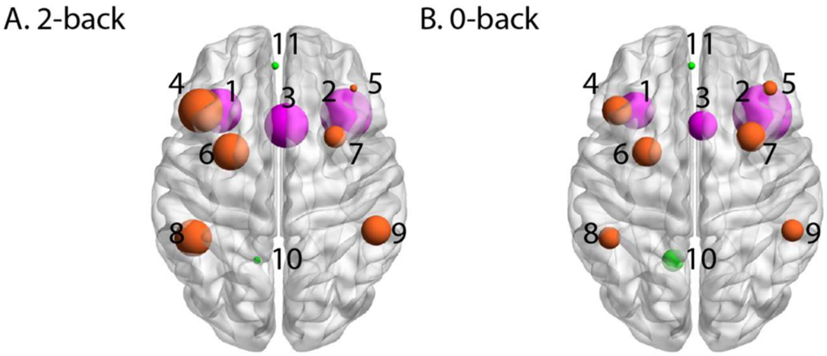

Supplementary Figure 18. Dynamic causal interactions between ROIs in Salience Network (SN), Frontal-Parietal Network (FPN) and Default Mode network (DMN): 1, left anterior insula (lAI); 2, right anterior insula (rAI); 3, dorsomedial prefrontal cortex (DMPFC); 4, left middle frontal gyrus (lMFG); 5, right middle frontal gyrus (rMFG); 6, left frontal eye field (lFEF); 7, right frontal eye field (rFEF); 8, left intraparietal lobule (lIPL); 9, right intraparietal lobule (rIPL); 10, posterior cingulate cortex (PCC) and 11, ventromedial prefrontal cortex (VMPFC) in (**A**) 2-back and (**B**) 0-back task conditions.

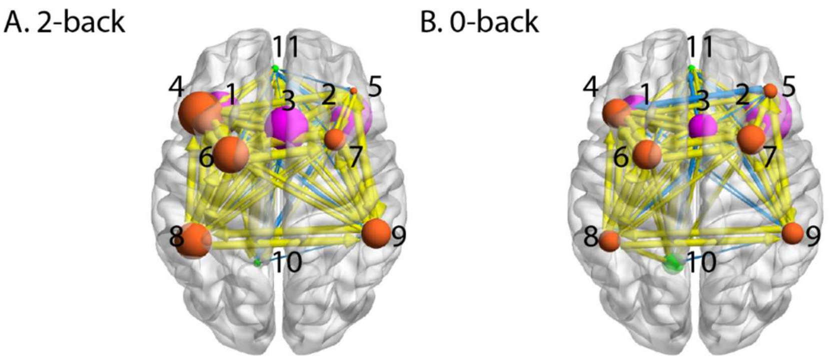

IV. Supplementary References

1. Pasqualetti F, Zampieri S, Bullo F. Controllability Metrics, Limitations and Algorithms for Complex Networks. *Ieee T Control Netw* **1**, 40-52 (2014).

2. Leitold D, Vathy-Fogarassy A, Abonyi J. Controllability and observability in complex networks - the effect of connection types. *Sci Rep* **7**, 151 (2017).

3. Sojoudi S, Doyle J. Study of the Brain Functional Network Using Synthetic Data. *Ann Allerton Conf*, 350-357 (2014).

4. Hespanha JP. *Linear systems theory*. Princeton Press (2009).

5. Summers TH, Cortesi FL, Lygeros J. On Submodularity and Controllability in Complex Dynamical Networks (vol 3, pg 91, 2016). *Ieee T Control Netw* **5**, 1503-1503 (2018).

6. Stephan KE, Kamper L, Bozkurt A, Burns GA, Young MP, Kotter R. Advanced database methodology for the Collation of Connectivity data on the Macaque brain (CoCoMac). *Philos Trans R Soc Lond B Biol Sci* **356**, 1159-1186 (2001).
