# Supplementary material for: Dynamic causal brain circuits during working memory and their functional controllability
Source: Nat Commun. 2021 Jun 29;12:3314. doi: 10.1038/s41467-021-23509-x (PMC8241851; doi:10.1038/s41467-021-23509-x)
Supplement: Supplementary file 3 — Reporting Summary [file 41467_2021_23509_MOESM3_ESM.pdf]

nature research

Corresponding author(s): Weidong Cai

Last updated by author(s): Mar 8, 2021

Reporting Summary

Nature Research wishes to improve the reproducibility of the work that we publish. This form provides structure for consistency and transparency in reporting. For further information on Nature Research policies, see our Editorial Policies and the Editorial Policy Checklist.

Statistics

For all statistical analyses, confirm that the following items are present in the figure legend, table legend, main text, or Methods section.

n/a	Confirmed	
☐	☒	The exact sample size (n) for each experimental group/condition, given as a discrete number and unit of measurement
☐	☒	A statement on whether measurements were taken from distinct samples or whether the same sample was measured repeatedly
☐	☒	The statistical test(s) used AND whether they are one- or two-sided<br>*Only common tests should be described solely by name; describe more complex techniques in the Methods section.*
☒	☐	A description of all covariates tested
☐	☒	A description of any assumptions or corrections, such as tests of normality and adjustment for multiple comparisons
☐	☒	A full description of the statistical parameters including central tendency (e.g. means) or other basic estimates (e.g. regression coefficient) AND variation (e.g. standard deviation) or associated estimates of uncertainty (e.g. confidence intervals)
☐	☒	For null hypothesis testing, the test statistic (e.g. F, t, r) with confidence intervals, effect sizes, degrees of freedom and P value noted<br>*Give P values as exact values whenever suitable.*
☒	☐	For Bayesian analysis, information on the choice of priors and Markov chain Monte Carlo settings
☒	☐	For hierarchical and complex designs, identification of the appropriate level for tests and full reporting of outcomes
☐	☒	Estimates of effect sizes (e.g. Cohen's d, Pearson's r), indicating how they were calculated

Our web collection on statistics for biologists contains articles on many of the points above.

Software and code

Policy information about availability of computer code

Data collection	We used the public Human Connectome Project dataset.
Data analysis	SPM12 was used for preprocessing fMRI data. MDS scripts are available from the Stanford SCSNL website (https://med.stanford.edu/scsnl/publications.html).

For manuscripts utilizing custom algorithms or software that are central to the research but not yet described in published literature, software must be made available to editors and reviewers. We strongly encourage code deposition in a community repository (e.g. GitHub). See the Nature Research guidelines for submitting code & software for further information.

Data

Policy information about availability of data

All manuscripts must include a data availability statement. This statement should provide the following information, where applicable:

- Accession codes, unique identifiers, or web links for publicly available datasets
- A list of figures that have associated raw data
- A description of any restrictions on data availability

The n-back working memory task fMRI data is accessible from the HCP database (https://db.humanconnectome.org/). All the simulation data are available from the authors.

Field-specific reporting

Please select the one below that is the best fit for your research. If you are not sure, read the appropriate sections before making your selection.

- [x] Life sciences
- [ ] Behavioural & social sciences
- [ ] Ecological, evolutionary & environmental sciences

For a reference copy of the document with all sections, see nature.com/documents/nr-reporting-summary-flat.pdf

Life sciences study design

All studies must disclose on these points even when the disclosure is negative.

Sample size	HCP data from 737 right-handed individuals (age: 22-36 years old, 413 female/324 male) were selected from a total 1200 subjects based on the following criteria: (1) participant had complete n-back task behavioral and fMRI data ; (2) range of head motion in any translational and rotational direction less than 1 voxel; (3) average scan-to-scan head motion less than 0.2 mm; (4) accuracies in 0-back and 2-back conditions greater than 50%; and (5) criterion (1) - (4) met in both sessions separately.
Data exclusions	(1) participant had complete n-back task behavioral and fMRI data ; (2) range of head motion in any translational and rotational direction less than 1 voxel; (3) average scan-to-scan head motion less than 0.2 mm; (4) accuracies in 0-back and 2-back conditions greater than 50%; and (5) criterion (1) - (4) met in both sessions separately.
Replication	All the main findings were replicated using subset samples, a different set of ROIs and normalized causal interaction weights.
Randomization	N/A
Blinding	N/A

Reporting for specific materials, systems and methods

We require information from authors about some types of materials, experimental systems and methods used in many studies. Here, indicate whether each material, system or method listed is relevant to your study. If you are not sure if a list item applies to your research, read the appropriate section before selecting a response.

Materials & experimental systems

n/a	Involved in the study	
x		Antibodies
x		Eukaryotic cell lines
x		Palaeontology and archaeology
x		Animals and other organisms
	x	Human research participants
x		Clinical data
x		Dual use research of concern

Methods

n/a	Involved in the study	
x		ChIP-seq
x		Flow cytometry
	x	MRI-based neuroimaging

Human research participants

Policy information about studies involving human research participants

Population characteristics	We did not use covariate analysis in this study.
Recruitment	We used all the data from the public human connectome project study and only excluded participants who have large head motion and poor behavioral performance.
Ethics oversight	Data acquisition for the HCP was approved by the Institutional Review Board of The Washington University in St. Louis (IRB # 201204036), and data were de-identified.

Note that full information on the approval of the study protocol must also be provided in the manuscript.

Magnetic resonance imaging

Experimental design

Design type	mixed block design
Design specifications	Subjects were presented with blocks of trials that consisted of pictures of faces, places, tools and body parts. Within each session, the 4 different stimulus types were presented in separate blocks. Furthermore, within each session, half of

the blocks are 2-back working memory tasks and half are 0-back working memory tasks. In the 2-back working memory task blocks, subjects were requested to determine whether the current stimulus matches the stimulus in two presentations of stimuli prior within the same block. In the 0-back working memory task blocks, subjects were requested to determine whether the current stimulus matches the target that was presented in the beginning of each block (cue). A 2.5 second cue indicates the task type (and target for the 0-back task) at the beginning of each block. Each of the two sessions contains 8 task blocks (10 trials of 2.5 seconds each, for 25 seconds) and 4 fixation ("rest") blocks (15 seconds). On each trial, the stimulus is presented for 2 seconds, followed by a 0.5 second inter-trial-interval (ITI).

Behavioral performance measures: Reaction time and accuracy

Acquisition

Imaging type(s): functional MRI

Field strength: 3T

Sequence & imaging parameters: multiband, gradient-echo planar imaging with the following parameters: TR=720ms; TE=33.1ms; flip angle=52°; field of view=280×180 mm; matrix=140×90; and voxel dimensions=2mm isotropic.

Area of acquisition: Whole brain

Diffusion MRI: ☐ Used ☒ Not used

Preprocessing

Preprocessing software: Raw fMRI data for both sessions were obtained from the HCP and underwent standard preprocessing steps, including realignment, slice-time correction, normalization and spatial smoothing with a Gaussian kernel of 6mm FWHM using SPM12.

Normalization: fMRI data was normalized using standard nonlinear transformation steps implemented in SPM12.

Normalization template: MNI152

Noise and artifact removal: 6 head motion parameters were regressed out.

Volume censoring: No volume censoring was done because a stringent head motion criterion was used for data inclusion

Statistical modeling & inference

Model type and settings: We used multivariate dynamic causal modeling on individual level of data and predictive model for both condition classification. Details of algorithm was provided in the method.

Effect(s) tested: We used paired t-test for between condition comparison with FDR correction.

Specify type of analysis: ☐ Whole brain ☒ ROI-based ☐ Both

Anatomical location(s): ROIs were made 6 mm radius spheres, whose centers were determined from a previous study.

Statistic type for inference (See Eklund et al. 2016): N/A

Correction: FDR correction was used for multiple comparison

Models & analysis

n/a	Involved in the study
☐	☒ Functional and/or effective connectivity
☐	☒ Graph analysis
☐	☒ Multivariate modeling or predictive analysis

Functional and/or effective connectivity: We used multivariate dynamical systems identification (MDSI) to investigate causal interactions.

Graph analysis: We determined the community structure of dynamic causal interactions in the 2-back and 0-back working memory task conditions using the Louvain algorithm

Multivariate modeling and predictive analysis: We used linear support vector machine and Lasso and Elastic-Net Regularized Generalized Linear Models for condition classification and 10-fold cross validation. The features are dynamic causal interactions between regions in salience, frontoaparietal and default mode networks. The label to be predicted is either '0-back' or '2-back' task condition.
